# WDR79 promotes aerobic glycolysis of pancreatic ductal adenocarcinoma (PDAC) by the suppression of SIRT4

**DOI:** 10.1515/med-2022-0624

**Published:** 2023-01-16

**Authors:** Wenke Yin, Xiaoyan Song, Yue Xiang

**Affiliations:** Department of Pathology, Institute of Basic Medicine and Forensic Medicine, North Sichuan Medical College, No. 55 Dongshun Road, Gaoping District, Nanchong, Sichuan, 637100, China; Department of Pathology, Affiliated Hospital of North Sichuan Medical College, Nanchong, Sichuan, 637000, China; Ultrasonography Lab, Nanchong Oriental Hospital, Nanchong, Sichuan, 637000, China

**Keywords:** Pancreatic adenocarcinoma, pancreatic ductal adenocarcinoma, WD repeat protein 79, aerobic glycolysis, SIRT4, UHRF1

## Abstract

Pancreatic cancer (PC) is an aggressive malignant disease. Pancreatic ductal adenocarcinoma (PDAC) is a main type of PDAC. The inhibition of aerobic glycolysis in PC cells is one of the approaches to treat PDAC. WD repeat protein 79 (WDR79) acts as a scaffold protein and is involved in several physiological processes. Since WDR79 affects the progression of several types of cancers, whereas its role in PDAC remains unclear. This study was aimed to investigate the role of WDR79 in the progression of PDAC and clarify the mechanism. We found that WDR79 was highly expressed in PDAC cells. Knockdown of WDR79 inhibited the growth as well as the motility of PDAC cells, while overexpression of WDR79 contributed to the growth and motility. The ablation of WDR79 restrained aerobic glycolysis of PDAC cells. Mechanically, we found that WDR79 depletion increased SIRT4 expression by suppressing UHRF1 expression, which counteracted the function of WDR79 in PDAC. We thought that WDR79 could serve as a target for treating PDAC.

## 1 Introduction

Pancreatic cancer (PC) is an aggressive malignant disease with a poor prognosis and has an overall 5-year survival rate of about 6% [1,2]. Pancreatic ductal adenocarcinoma (PDAC) is a main type of PDAC. The poor prognosis of PDAC is attributed to its early metastasis and invasion [3]. In addition, PDAC tumors exhibit metabolic changes to meet the need for unlimited proliferation [4,5,6]. Among them, metabolomic studies have shown that the Warburg effect (i.e., aerobic glycolysis) occurred in PC tissues and influenced its malignant progression [7].

WD repeat protein 79 (WDR79) (also known as WRAP53/TCAB1) acts as a scaffold protein and is involved in the physiological processes of telomerase localization, telomere and Cajal body assembly, and DNA double-strand break repair [8,9,10]. Abnormal expression of WDR79 was found in several types of cancers including colorectal cancer, head and neck cancer, breast cancer, ovarian cancer, etc. [8,11,12]. WDR79 was also overexpressed in esophageal cancer and promotes the proliferation of esophageal cancer cells [13]. WDR79 regulates USP7 activity, thereby promoting the ubiquitination and degradation of P53 and MDM2 and promoting the cell proliferation of non-small-cell lung cancer [14]. These studies suggest that WDR79 plays a key role in cancer progression.

The Cancer Genome Atlas (TCGA) analysis shows that WDR79 is highly expressed in PC patients. In addition, WDR79 can increase the stability and expression of UHRF1 [15]. UHRF1 is an oncogene that was overexpressed in many cancers and promotes cancer progression. Importantly, UHRF1 is overexpressed in PC and promotes the aerobic glycolysis process and proliferation of PC by inhibiting SIRT4 expression [16]. Among them, SIRT4 is a mitochondrial negative regulator of aerobic glycolysis, and its high expression can inhibit tumor proliferation or increase the sensitivity of tumor cells to chemotherapy drugs in PC [16,17]. In addition, the UHRF1/SIRT4/HIF1α axis regulated the glycolysis process of PDAC cells, thereby promoting their malignant proliferation [16]. It is interesting to uncover whether WDR79 could affect PDAC progression via SIRT4.

In this study, we investigated the expression of WDR79 in PC and unraveled the possible mechanisms. Our data confirmed that WDR79 could act as a potential target for treating PC.

## 2 Materials and methods

### 2.1 Bioinformation analysis

We conducted bioinformation analysis through GEPIA (http://gepia.cancer-pku.cn/) to analyze expression level and survival in TCGA database.

### 2.2 Antibodies and plasmids

**Table:** 

Antibody	Dilution	Company, article number
WDR79	1:500	ab224444, Abcam
E-cadherin	1:1,000	ab40772, Abcam
N-cadherin	1:500	ab245117, Abcam
SIRT4	1:500	ab231137, Abcam
UHRF1	1:500	ab213223, Abcam
GLUT1	1:500	ab115730, Abcam
HK2	1:500	ab209847, Abcam
LDHA	1:500	ab52488, Abcam
GAPDH	1:3,000	ab9485, Abcam

The p-Super shRNA plasmids were bought from Addgene and constructed by our lab. The plasmids including pcDNA3.1 and pcDNA3.1-WDR79 were constructed in our lab.

### 2.3 Cell culture and transfection

The HPAC, Capan-1, SW1990, and CFPAC-1, and normal pancreatic cell line such as hTERT-HPNE, were purchased from American Type Culture Collection and maintained in Dulbecco’s modified Eagle’s medium (with 10% of fetal bovine serum) at 37°C in a 5% CO_2_ incubator.

### 2.4 Immunoblot assay

Samples were lysed by RIPA buffer (9800; Cell Signaling). All the cell and tissue samples were isolated to extract the proteins, separated by 10% SDS-polyacrylamide gel electrophoresis, then transferred onto polyvinylidene fluoride membranes and blocked with 10% fat-free milk in TBS with Tween-20 buffer. All membranes were subsequently incubated with the indicated primary antibodies targeted a series of proteins for 2 h. Subsequently, the membranes were incubated with secondary antibodies for 1 h. Blots were then measured.

### 2.5 qPCR

qPCR was performed with the SYBR-Green Master Mix (Roche, USA) and respective primers. The used primers were listed as below: WDR79: F: 5′-AATCAGCGCATCTACTTCGAT-3′, R: 5′-AAATCGAAGTAGATGCGCTGA-3′, GAPDH: F: AGAAGGCTGGGGCTCATTTG, R: AGGGGCCATCCACAGTCTTC′.

### 2.6 Cell counting kit-8 (CCK-8) assay

PDAC cells were plated into the 96-well plates with a density of 1,000 cell per well and maintained for 24, 48, 72, and 96 h. Cells were then incubated with CCK-8 for 4 h and the OD value at the wavelength of 450 nm was measured.

### 2.7 Transwell assays

Cells transfected with indicated plasmids were plated into the upper chamber of transwell chambers in culture medium without serum. Then, medium containing 10% FBS was added into the bottom to stimulate motility. After 24 h, cells in the upper chamber were removed, and the remaining cells were fixed, as well as stained with 0.2% crystal violet.

### 2.8 Glucose intake test

The glycolysis levels of cells were detected according to the glucose intake (ab136955), which was measured by the corresponding kits bought from Abcam according to the relevant instructions.

### 2.9 Oxygen consumption rate (OCR) and extracellular acidification rate (ECAR) test

OCR and ECAR were detected by an XF24 Extracellular Flux Analyzer (Seahorse Bioscience, Billerica, MA, USA) following the manufacture’s guidelines. OCR and ECAR measurement was performed after the exchange of medium for 90 min. The inhibitors of electron transport chain and oxidative phosphorylation (OXPHOS), including oligomycin A (1 μM), CCCP (1.5 μM), rotenone (0.5 μM), and antimycin A (0.5 μM), were injected.

### 2.10 Statistics

GraphPad 5.0 software was used for the statistical analysis. Data were expressed as mean ± SEM. Student’s *t*-test was used for the comparisons, and *p* < 0.05 was considered significant difference.

## 3 Results

### 3.1 WDR79 was highly expressed in human PDAC tissues and cells

**Figure 1:**
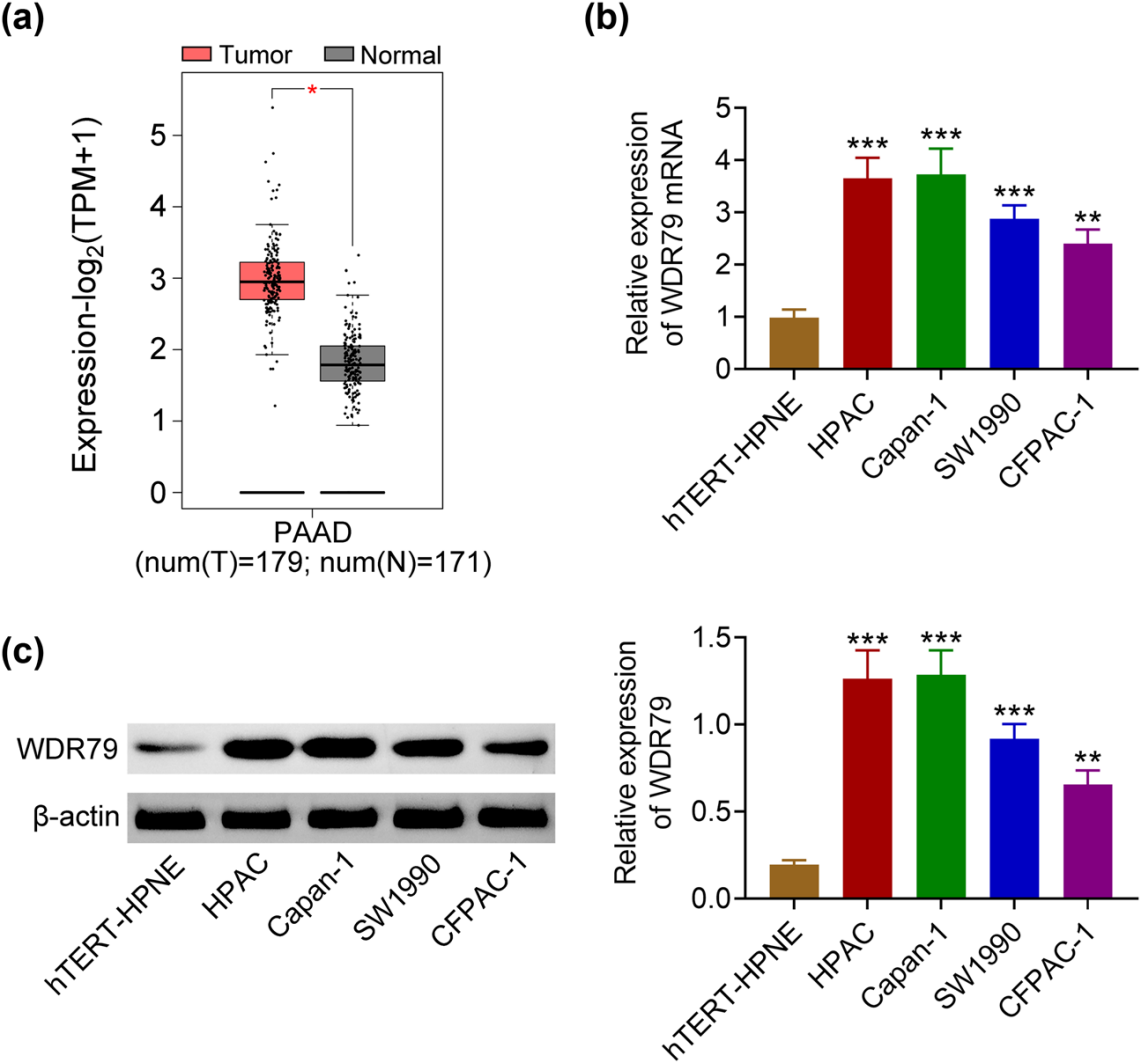
WDR79 was highly expressed in pancreatic cancer tissues and cells. (a) TCGA database showed the expression of WDR79 in pancreatic cancer tissues and normal tissues. (b) qPDACR assays showed the mRNA levels of WDR79 in cell lines including hTERT-HPNE, HPAC, Capan-1, SW1990, and CFPAC-1. (c) Immunoblot showed the protein levels of WDR79 in cell lines including hTERT-HPNE, HPAC, Capan-1, SW1990, and CFPAC-1. **p < 0.05, ***p < 0.01. WDR79 vs control; ^#^ *p* < 0.01, ^##^ *p* < 0.05, ^###^ *p* < 0.01. shWDR79 vs shControl.

We first detected the expression of WDR79 in tumor tissues and cells. We detected the expression of WDR79 in PDAC tissues (*n* = 179) and normal tissues (171) according to the TCGA database. We found that the expression of WDR79 was high in PDAC tissues of patients (Figure 1a). Subsequently, we detected the mRNA levels and protein levels of WDR79 in PDAC cells including HPAC, Capan-1, SW1990, and CFPAC-1, and normal pancreatic cell line hTERT-HPNE. The qPCR and immunoblot results revealed that the expression of WDR79 was high in both mRNA and protein levels in PDAC cells compared to normal pancreatic cells (Figure 1b and c). Therefore, WDR79 was highly expressed in human PDAC tissues and cells.

### 3.2 WDR79 depletion suppressed viability as well as the motility of PDAC cells

**Figure 2:**
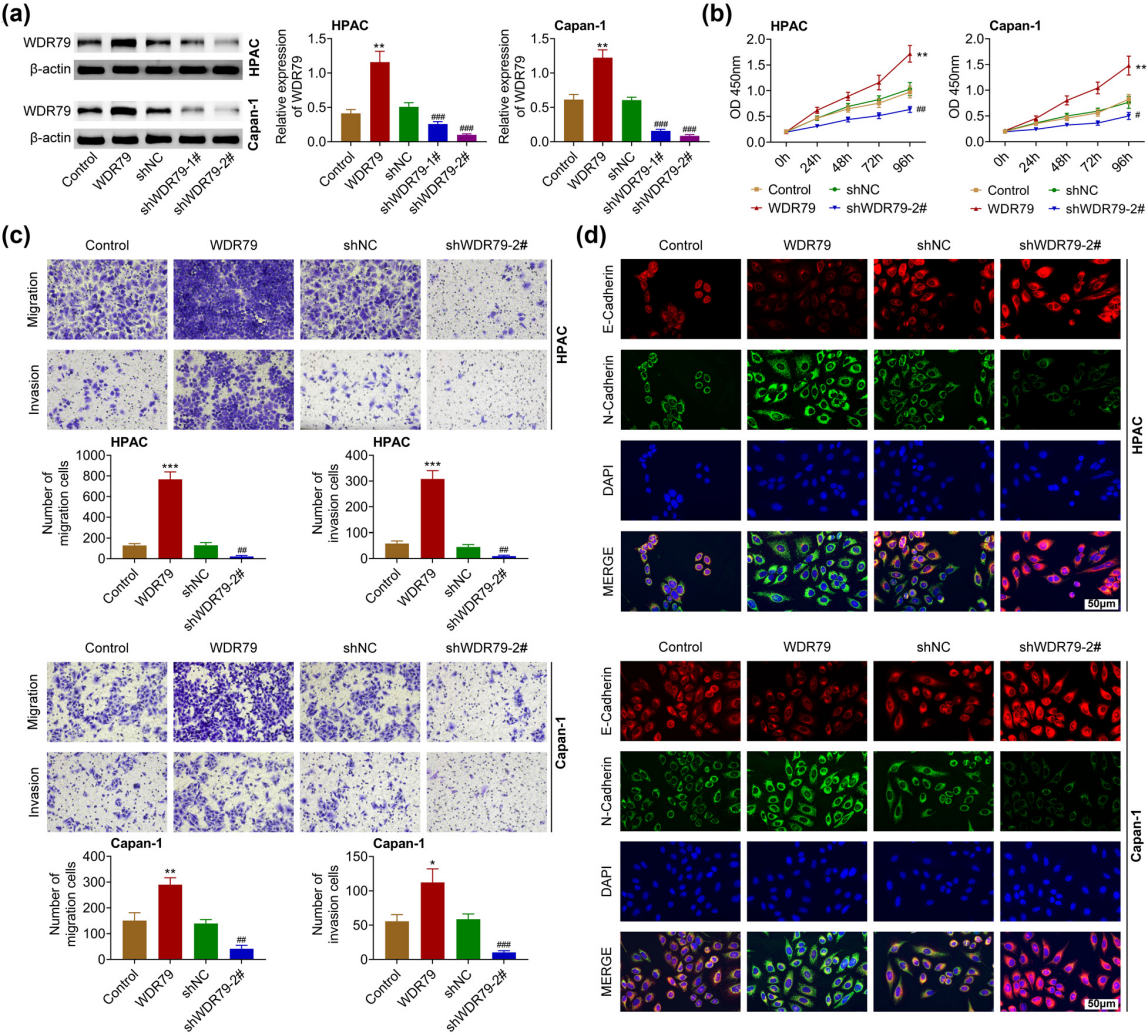
Effects of WDR79 on the viability and motility of pancreatic cancer cells. (a) Immunoblot showed the expression of WDR79 in HPAC and Capan-1 cells upon the indicated transfection. (b) CCK-8 assays showed the OD value at 450 nm wavelength of HPAC and Capan-1 cells upon the indicated transfection at 24, 48, 72, and 96 h. (c) Transwell showed the invasive HPAC and Capan-1 cells upon the transfection of indicated plasmids or shRNAs. (d) Immunoblot showed the expression of E-cadherin and N-cadherin in HPAC and Capan-1 cells upon the indicated transfection. **p < 0.01, ***p < 0.01, WDR79 vs control; ^#^ *p* < 0.01, ^##^ *p* < 0.05, ^###^ *p* < 0.01. shWDR79 vs shControl.

Subsequently, we detected the effects of WDR79 on the viability as well as the motility of PDAC cells. The plasmids and shRNAs of WDR79 were transfected into two types of PDAC cells, including HPAC and Capan-1, to alter the expression of WDR79 in PDAC cells. Immunoblot results revealed the transfection of WDR79 plasmids obviously increased its expression in HPAC and Capan-1 cells, whereas the transfection of WDR79 shRNAs (shRNA 1# and 2#) significantly suppressed the expression of WDR79 in PDAC cells (Figure 2a).

CCK-8 assays showed thatWDR79 overexpression promoted the viability of HPAC and Capan-1 cells, and its depletion suppressed the viability of cells (Figure 2b). Transwell assays suggested that WDR79 overexpression contributed to the migration of HPAC and Capan-1 cells, whereas its knockdown decreased invasive cell numbers of PDAC cells (Figure 2c). Subsequently, immunostaining assays were performed to detect the expression of E-cadherin and N-cadherin, which were two biomarkers of cell motility. We noticed that WDR79 overexpression decreased expression of E-cadherin and increased N-cadherin expression, whereas its depletion (sh 2#) increased E-cadherin and decreased N-cadherin expression in HPAC and Capan-1 cells (Figure 2d). Therefore, WDR79 depletion suppressed viability as well as the motility of PDAC cells.

### 3.3 WDR79 knockdown inhibited aerobic glycolysis of PDAC cells

**Figure 3:**
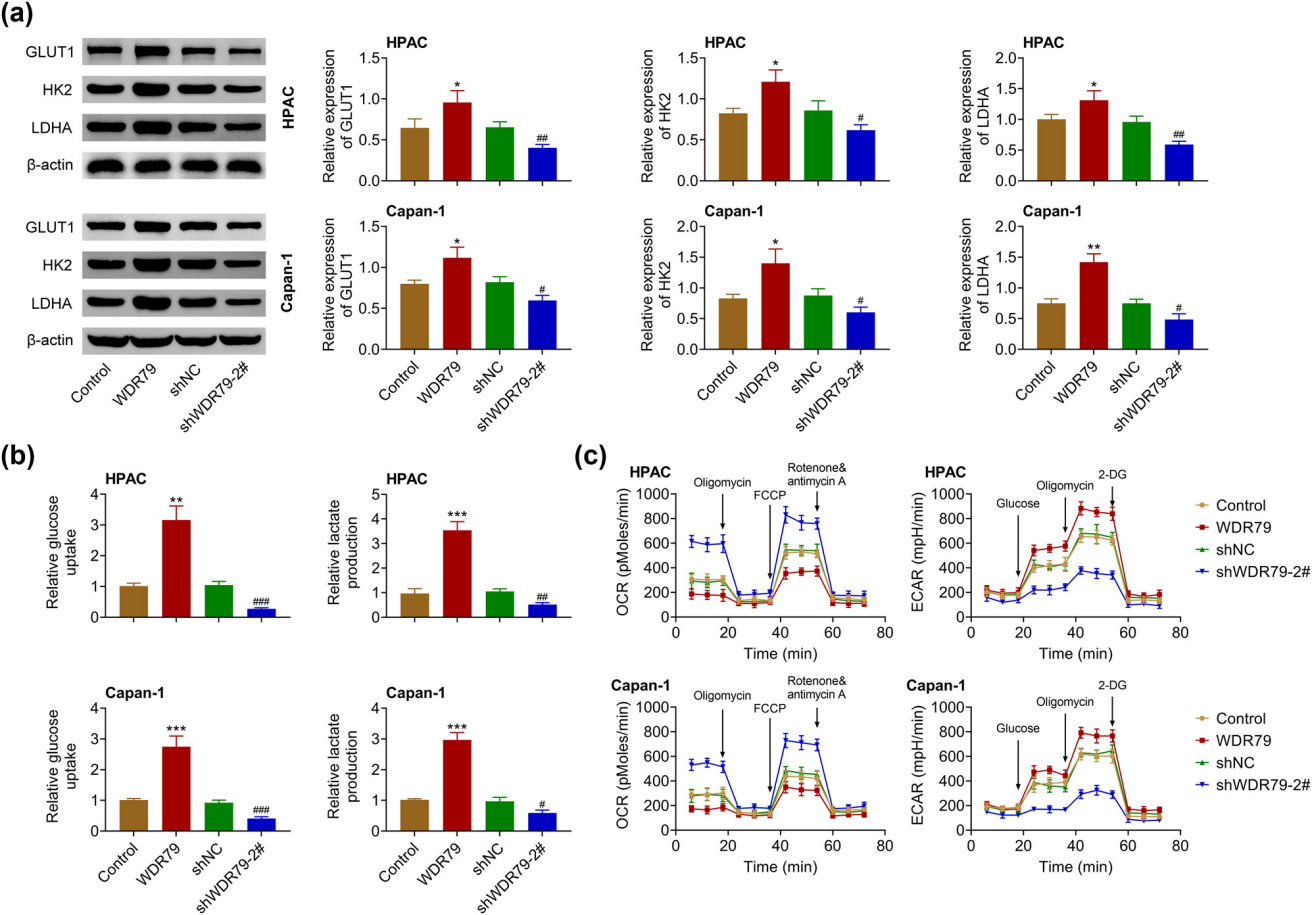
WDR79 knockdown inhibited aerobic glycolysis of pancreatic cancer cells. (a) Immunoblot showed the expression of GLUT1, HK2, LDHA in HPAC, and Capan-1 cells upon the indicated transfection. (b) Glucose uptake assays showed the relative glucose uptake capacity of HPAC and Capan-1 cells upon the indicated transfection. (c) OCR and ECAR levels of HPAC and Capan-1 cells upon the indicated transfection. *p < 0.05, **p < 0.01, ***p < 0.01, WDR79 vs control; ^#^ *p* < 0.01, ^##^ *p* < 0.05, ^###^ *p* < 0.01. shWDR79 vs shControl.

Then, the effect of WDR79 on the aerobic glycolysis of PDAC cells was detected. Immunoblot assays were carried out to detect the expression of markers of aerobic glycolysis, including GLUT1, HK2, as well as LDHA in HPAC and Capan-1 cells upon the overexpression and depletion of WDR79. We found that WDR79 overexpression increased the expression of GLUT1, HK2, as well as LDHA, and the depletion of WDR79 suppressed the expression of these proteins in HPAC and Capan-1 cells (Figure 3a). We subsequently performed glucose uptake assays, and the data confirmed that WDR79 overexpression promoted the glucose uptake in HPAC and Capan-1 cells, whereas its depletion suppressed the glucose uptake (Figure 3b). We then performed OCR and ECAR assays and found that WDR79 overexpression contributed to the OCR and suppressed ECAR in HPAC and Capan-1 cells, which was reversed by the depletion of WDR79 (Figure 3c). Therefore, WDR79 knockdown inhibited the aerobic glycolysis of PDAC cells.

### 3.4 Depletion of WDR79 increased SIRT4 expression by suppressing UHRF1

**Figure 4:**
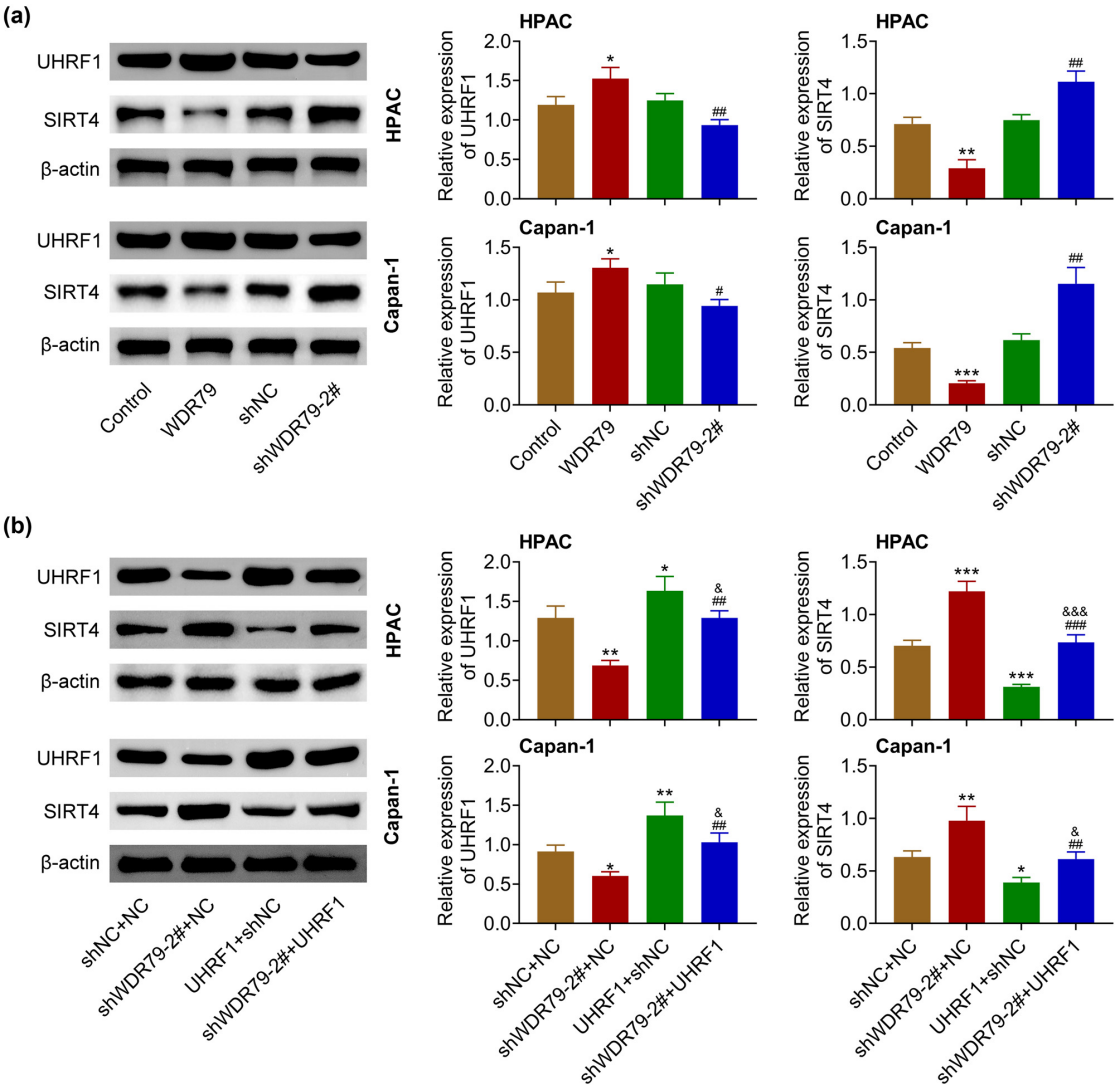
WDR79 depletion increased SIRT4 expression by inhibiting UHRF1 in pancreatic cancer cells. (a) Immunoblot showed the expression of UHRF1 and SIRT4 in HPAC and Capan-1 cells upon the indicated transfection. (b) Immunoblot showed the expression of UHRF1 and SIRT4 in HPAC and Capan-1 cells upon the indicated transfection. *p < 0.05, **p < 0.01, ***p < 0.01, WDR79 vs control; ^#^ *p* < 0.01, ^##^ *p* < 0.05, ^###^ *p* < 0.01. shWDR79 vs shControl. ^&^ *p* < 0.05, ^&&&^ *p* < 0.01, UHRF1 vs NC.

Immunoblot assays revealed that WDR79 overexpression increased the expression of UHRF1 and decreased the expression of SIRT4 in HPAC and Capan-1 cells (Figure 4a). The depletion of WDR79 suppressed UHRF1 expression and increased SIRT4 expression in HPAC and Capan-1 cells (Figure 4a). Subsequently, we performed rescue assays. Our data confirmed that UHRF1 overexpression reversed the increase of SIRT4 induced by WDR79 depletion in both HPAC and Capan-1 cells (Figure 4b). We therefore thought that ablation of WDR79 increased SIRT4 expression by suppressing UHRF1 expression.

### 3.5 Depletion of SIRT4 expression reversed the effect of WDR79

**Figure 5:**
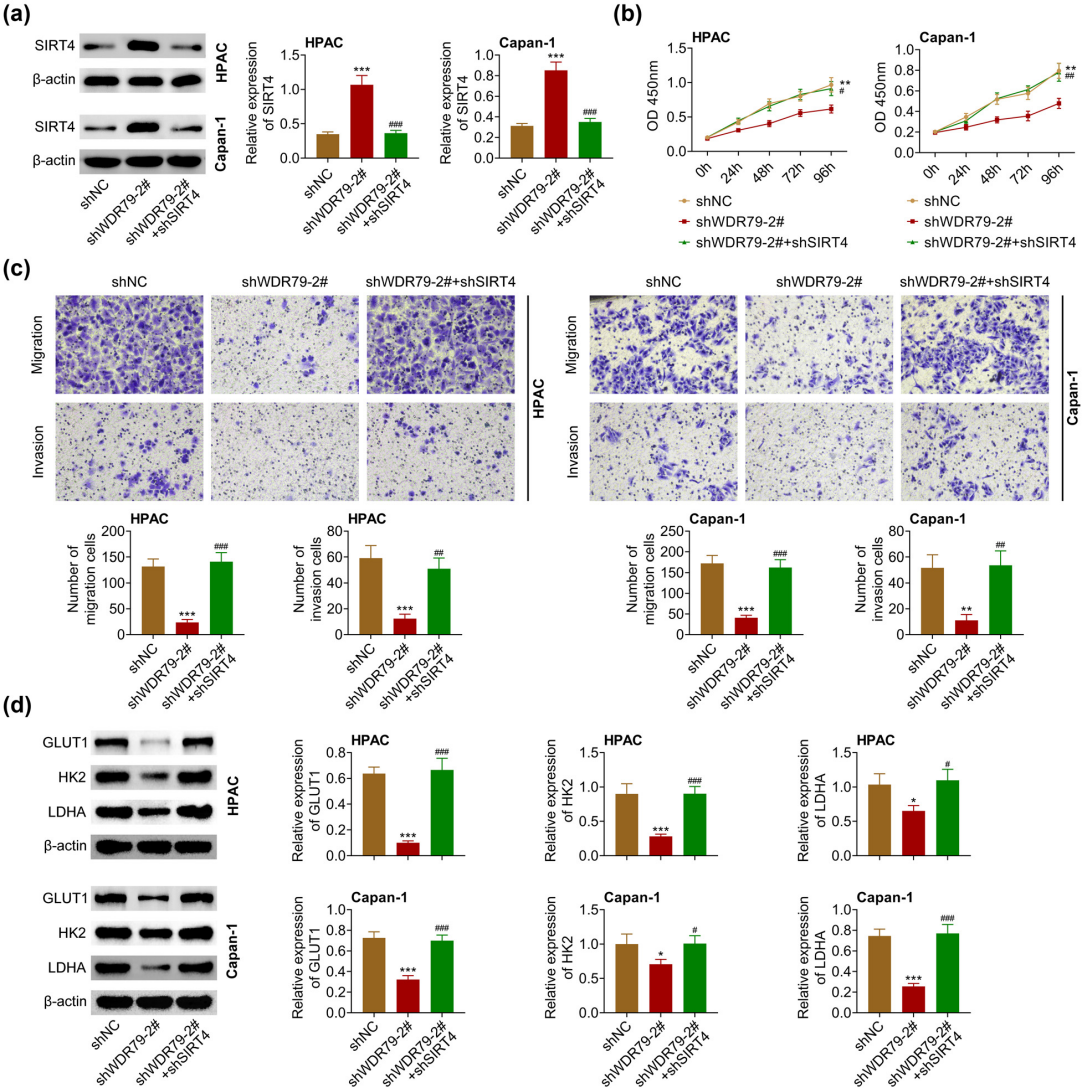
Depletion of SIRT4 expression reversed the effects of WDR79. (a) Immunoblot showed the expression of SIRT4 in HPAC and Capan-1 cells upon the indicated transfection. (b) CCK-8 assays showed the OD value at 450 nm wavelength of HPAC and Capan-1 cells upon the indicated transfection at 24, 48, 72, and 96 h. (c) Transwell showed the invasive pancreatic cancer cells upon the transfection of indicated plasmids or shRNAs. (d) Immunoblot showed the expression of E-cadherin and N-cadherin in HPAC and Capan-1 cells upon the indicated transfection. *p < 0.05, **p < 0.01, ***p < 0.01, shWDR79 vs shControl; ^#^ *p* < 0.01, ^##^ *p* < 0.05, ^###^ *p* < 0.01. shWDR79 + shSIRT4 vs shWDR79 + shControl.

We then performed immunoblot assays to detect the effects of SIRT4 on the anti-tumor effect of WDR79 in PDAC. We found that WDR79 overexpression decreased SIRT4 expression, whereas SIRT4 shRNA transfection increased SIRT4 expression in WDR79 overexpressed HPAC and Capan-1 cells (Figure 5a). We further performed CCK-8 assays and found that WDR79 depletion suppressed the proliferation of PDAC cells, which was reversed by SIRT4 depletion in WDR79 depleted cells (Figure 5b). Furthermore, transwell assay results suggested that WDR79 depletion suppressed cell motility, which was also reversed by SIRT4 depletion (Figure 5c). In addition, WDR79 depletion suppressed the expression of GLUT1, HK2, as well as LDHA, and the depletion of SIRT4 further reversed the inhibition expression of these proteins (Figure 5d). Therefore, depletion of SIRT4 expression reversed the anti-tumor effect of WDR79.

## 4 Discussion

Up to now, there have been a lot of studies on the pathogenesis of PC, and it is still needed to improve the treatment method [3]. Currently, several therapeutic targets have been discovered, but drugs developed from these therapeutic targets have little significant effect [18]. To further improve patient survival, a better understanding of its heterogeneity and pathogenesis is needed, as well as further identification of more effective molecular targets [19]. In this study, we revealed that a member of WDR family, WDR79, was highly expressed in human PDAC tissues as well as cells. WDR79 depletion inhibited the growth, motility, and restrained aerobic glycolysis of PDAC cells. We found that WDR79 depletion increased SIRT4 expression by suppressing UHRF1 expression in PC and thought it could act as a molecular target for treating PDAC.

Through a series of *in vitro* assays, including CCK-8, transwell, and immunoblot, we confirmed the overexpression of WDR79 contributed to the growth and motility of PDAC cells. Furthermore, the immunoblot assay glucose intake, OCR, as well as ECAR assays revealed the effects of WDR79 on the aerobic glycolysis of PDAC cells. Collectively, we thought that WDR79 could affect the glycolysis of PDAC cells, thereby affecting cell viability, motility, and tumor growth. The role of WDR79 in cancer progression has been investigated [8,10]. WDR79 was associated with its mutation as well as prognosis of lung cancer [8,12]. Its mutation in breast cancer has also been reported [8].

PDAC is characterized by a high glycolysis rate that ensures cancer cell survival, resulting in a nutrient deficient and highly hypoxic microenvironment [20]. The glycolysis process of PDAC cells produces a large number of substrates and promotes PDAC cell growth as well as metastasis through the interaction of glycolysis enzyme, thereby supporting tumor growth [21]. In addition, the core enzymes and intermediates of aerobic glycolysis influence the growth and metastasis of PDAC by participating in EMT as well as proliferation signal transduction or epigenetic regulation [22,23]. These studies confirmed the key role of aerobic glycolysis and the related proteins in the progression of PDAC. Our data further confirmed that targeting the aerobic glycolysis was a promising method to combat PDAC.

Our data further confirmed that the depletion of WDR79 increased SIRT4 expression by suppressing UHRF1. UHRF1 is an epigenetic modifier whose SRA domain can specifically recognize and bind to CpG sites of hemimethylation to recruit DNMT1 to DNA and ensure the stability of DNA methylation [24,25]. It is overexpressed in some types of PC, but its precise role is unclear. One study showed that the UHRF1/SIRT4/HIF1α axis regulated the glycolysis process of PDAC cells, thereby promoting their malignant proliferation [16]. In this study, we confirmed that WDR79 could mediate the expression of UHRF1 and SIRT4 and affected the aerobic glycolysis of PDAC cells. In addition, the regulatory role of SIRT4 in the glycolysis of different types of cancers has been widely demonstrated.

In summary, we revealed that WDR79 was highly expressed in PDAC cells as well as tissues. WDR79 knockdown inhibited the growth, motility, and restrained aerobic glycolysis of PDAC cells. We found that WDR79 depletion increased SIRT4 expression by suppressing UHRF1 expression, and inhibition of SIRT4 expression reversed the anti-tumor effect of WDR79 in PC. We thought that WDR79 could serve as a target for treating PDAC.
